# Prevalence of Anaemia and Associated Risk Factors among Children in North-western Uganda: A Cross Sectional Study

**DOI:** 10.1186/s12878-017-0081-0

**Published:** 2017-07-03

**Authors:** Ismail Dragon Legason, Alex Atiku, Ronald Ssenyonga, Peter Olupot-Olupot, John Banson Barugahare

**Affiliations:** 1 School of Postgraduate Studies, Uganda Christian University, Mukono, Uganda; 2 Kuluva Hospital, Arua, Uganda; 3 School of Public Health, Makerere College of Health Sciences, Kampala, Uganda; 4 Faculty of Health Sciences, Busitema University, Mbale, Uganda; 5 Faculty of Science and Education, Busitema University, Tororo, Uganda

**Keywords:** Prevalence, Anaemia, Risk factors, Children, North-western Uganda

## Abstract

**Background:**

Despite the public health significance of anaemia in African children, its broader and often preventable risk factors remain largely under described. This study investigated, for the first time, the prevalence of childhood anaemia and its risk factors in an urban setting in Uganda.

**Methods:**

A total of 342 children were enrolled. Venous blood samples were collected in EDTA tubes and analyzed using Symex 500i (Symex Corp. Japan). Stool and urine samples were analyzed according to established standard methods. Anthropometric indicators were calculated according to the CDC/WHO 1978 references. Ethical approval was granted.

**Results:**

Categorically, the prevalence of anaemia was; 37.2, 33.3 and 11.8% among children aged 1–5 years, 6–11 years and 12–14 years respectively. Overall anaemia prevalence was 34.4%. The risk of anaemia was higher among males than females [(OR = 1.3, 95% CI = 0.8, 2.1), *P* = .22]. Malaria was associated with a 1.5 times risk of anaemia though not statistically significant in the multivariate analysis (*P = .19)*. Maternal parity <5 (*P* = .002), and stunting [(OR = 2.5, 95% CI = 1.3, 4.7), *P* = .004] were positively associated with anaemia. There was a positive correlation between household size and income (*Pearson X* ^*2*^ *= 22.96; P = .001)*, implying that large families were of higher socioeconomic status.

**Conclusions:**

This study demonstrates that anaemia is more prevalent in the under-5 age. The risk factors are stunting and low maternal parity. Interventions that address nutritional deficiencies in both pre-school and school children are recommended. Malaria and helminthiasis control measures counter the risk of anaemia. Further studies are required to investigate the association between maternal parity and anaemia found in this study.

## Background

Anaemia remains a major public health problem in young children particularly in the developing world [1]. Over 273 million children under – five, suffer from anaemia worldwide [2]. The Sub-Saharan Africa is one of the most affected regions - with more than half (53.8%) of children under - 5 years old suffering from anaemia [2]. In Uganda, some hospital-based data is available, however, there is a paucity of data on anaemia in school children and community based surveys. Nevertheless, in a few of the studies, anaemia prevalence ranges from 38 to 46% [3, 4]. In a more recent community pilot survey conducted in Arua district (same study area) hemoglobin <11.0 g/dl was observed in nearly half of the children less than 16 years enrolled (unpublished data).

Recent descriptions have shown that the aetiology of anaemia is multi-factorial with severe micronutrient deficiencies playing a major role [5], as opposed to earlier data describing the condition as being caused mainly by infectious agents, folate and iron deficiencies [2]. This calls for new approaches in prevention and management of childhood anaemia. Among the well described consequences are impaired physical growth [6], immune alterations and increased susceptibility to infections [7], impaired motor development leading to reduced cognitive ability [8–10], poor school performance [11] and short or long term mortality in acute severe cases [5]. Significant in the pathogenesis of anaemia is the fact that it is detrimental even before symptoms become apparent hence, the need to identify the risk factors for timely and effective management strategies.

In malaria endemic areas, the risk of anaemia is particularly important [12, 13]. Moreover, asymptomatic malaria infections often labelled carrier states, contribute to substantial development of anaemia even though mortality in this category is <1% [14, 15]. Infections with gastrointestinal helminthes and Schistosomes have also been shown to significantly contribute to the risk of childhood anaemia in the East African region [16, 17]. Despite the public health significance of anaemia in African children, its broader and often preventable risk factors remain largely under described. The aim of the study was to determine the prevalence of anaemia and associated risk factors among children aged 1–14 years in an urban setting.

## Methods

### Study design and setting

We conducted a cross sectional study among children aged 1–14 years in 20 local administrative units (villages) of Arua municipality in Arua district, North-western Uganda. The study was conducted between March and May 2016. A two-stage cluster-sampling was used in the survey. The first stage involved selection of villages. A probability proportional-to-number was applied to select the villages from each cluster. The second stage involved selection of households using Village health Team (VHT) registers.

### Inclusion/exclusion criteria

Children were eligible if they; were at least 1 year old but not more than 14 years old and had been living in the study area for at least 3 months prior to the study. Children were excluded if they had a history of anti malarial or helminthes treatment 2 weeks prior to the study, were not usual residents and their parents did not consent.

### Data collection

The data collection tool was developed based on the national survey questionnaire and was administered to the consenting parents/caregivers of the prospective enrolees. Height was measured using Charder HM200P- portable stadiometer (precision 0.1 cm) with child head positioned according to the Frankfurt plane. Weight was measured using Taylor digital floor scale with the child standing. Children who were unable to stand on their own were held on the scale and then difference in weight subtracted. Nutritional status was assessed and stratified by age and sex.

Up to 4 ml of venous blood was collected into EDTA vacutainer for complete blood count (CBC) performed on a 5-Diff automated analyzer, Symex 500i, Symex Corp. Japan. Typing of anaemia was by morphological examination of peripheral blood films and results were compared with MCV, MCHC and RDW values obtained on an automated analyzer. Reference data published by Lugada et al., was used to interpret the red cell indices [18]. Anaemia status was defined, adjusted for altitude as recommended by WHO [19]; Hb < 11.0 g/dl for age 6 months to 5 years, Hb < 11.5 g/dl for age 6 to 11 years and Hb < 12.0 g/dl for age 12 to 14 years. The severity of anaemia was classified based on the WHO scheme as mild (Hb ≥ 11 but less than normal), moderate (Hb between 8 and 10.9 g/dl) and severe (Hb < 8).

Thick and thin blood slides were prepared, stained with Giemsa and examined microscopically for malaria parasites. Malaria was excluded when thick smears were reported negative after examining 100 fields under ×100 oil immersion objective. The species of infecting *Plasmodium* parasite was determined using the thin blood film. Where it was not possible, a rapid malaria test kit with multiple species detection was used to identify the species. Children were provided with a pair of universal containers, a plastic bag, a clean non-disinfectant impregnated disposable towel and tissue paper in order to collect good quality stool samples and urine. Children above 7 years of age were instructed to lay the disposal kitchen towel and defecate on it. With the applicator stick or spoon attached to the cap of the stool container, pick about 2 g of sample and put into the container and deliver immediately to the laboratory. For younger children parents were given the same instructions above to collect stool samples. Intestinal helminthes were detected microscopically by wet preparation and Kato-Katz technique [20]. Urine specimens were first centrifuged at 1000 g for 5 min and sediment examined microscopically for Schistosome eggs. HIV screening was done in accordance to Uganda ministry of health guidelines. Plasma obtained from centrifuged whole blood was then used for testing HIV infection.

### Statistical analysis

The data was entered in EPi Info software version 3.3.2 (Centre for Diseases Control and prevention, USA) and analysed using Stata version 12 (Stata Corp., Texas). Proportions, means and medians were computed for the demographic characteristics. Pearson chi-square or Fisher’s test and analysis of variance (ANOVA) were calculated at bivariate analysis. Multivariate analysis was performed using a logistic regression model utilizing odds ratios to quantify the association and two-sided *P*-values to determine statistical significance. Interaction and confounding at a 10% cut off was assessed during the model fitting. The 95% confidence interval was determined and factors with a *P*-value <0.05 were considered significant.

Nutritional status was assessed and stratified by age and sex. Weight-for-age, height-for-age, and weight-for-height Z-scores were calculated based on CDC/WHO 1978 reference data using Epi Info version 3.3.2. Z-scores <−2 SD were indicative of underweight, stunting and wasting respectively [20].

## Results

Out of the 342 children enrolled, 183 (53.5%) were males and 159 (46.5%) females. Pre-school children (1-5 years) comprised 57.3% of the overall sample size. The mean age was 5.6 years and median age was 5.1 years. Children lived in households with an average of 7.3 members. The homes had an average of two rooms dedicated for sleeping. Business (commercial units) is the most common occupation (37.7%). Most homesteads (98%) had either a latrine or toilet for family use and this level of sanitation is above the average reported nationally [19]. Only 9.4% of the households were less than 0.25 km from a perennial water source. Mosquito bed net usage was reported by 93.9% of the households and 95.3% of the children reported having slept under bed net throughout the week before the study. Nearly all children (98.8%) lived in households that had access to an “improved” drinking water source (tap water, borehole, protected well or spring, bottled water). Primary school education is most common level of the children’s mothers (*N* = 195, 57.0%). Whereas, a majority of the children’s fathers (*N* = 209, 61.1%) had at least a secondary school education, only 110 (32.2%) females had attained similar levels of education. Only 12.6% of the households had difficulty satisfying their food needs and 88.8% reported having at least three meals per day. 23.7% (*N* = 81) of the households did not consume meat regularly. The baseline characteristics of the study population are included in Table 1.

**Table 1:** Showing some baseline characteristics of the study population

Variable	Age group in Years *n* = 342			Overall
	1–5 196 (57.3)	6–11 129 (37.7)	12–14 17 (5.0)	
Sex				
Male	111 (60.7)	66 (36.1)	6 (3.3)	183
Female	85 (53.5)	63 (39.6)	11 (6.9)	159
Age: mean (SD)	3.4 (1.4)	8.0 (1.6)	12.9 (0.9)	5.6 (3.1)
Religion				
Protestant	27 (51.9)	20 (38.5)	5 (9.6)	52
Islam	130 (63.4)	67 (32.7)	8 (3.9)	205
Catholic	39 (45.9)	42 (49.4)	4 (4.7)	85
Mother’s level of education				
No formal education	16 (43.2)	20 (54.1)	1 (2.7)	37
Primary	108 (55.4)	76 (39.0)	11 (5.6)	195
Secondary or above	72 (65.4)	33 (30.0)	5 (4.6)	110
Household size: mean (SD)	6.8 (3.4)	7.8 (4.6)	9.1 (2.9)	7.3 (4.0)
Household’s main source of income				
Employment/Salary	40 (50.6)	33 (41.8)	6 (7.6)	79
Agriculture related	6 (60.0)	4 (40.0)	0 (0)	10
Property income	19 (73.1)	6 (23.1)	1 (3.9)	26
Business enterprise	73 (56.6)	51 (39.5)	5 (3.9)	129
Other	58 (59.2)	35 (35.7	5 (5.1)	98
Monthly household income’000				
Median(IQR)	233.5 (150–300)	240(150–300)	300 (210–450)	240 (150–300)

Blood samples were collected from 332 (97.1%), stool from 291 (85.1%) and urine samples from 317 (92.7%). The urine sediments were microscopically examined for Schistosome eggs. Complete blood counts including hemoglobin measurement were available for 329 (96.2%) children. Overall, 14.2% of the children tested positive for malaria, 0.3% HIV positive and 15.5% had stool parasites detected in their samples. Malaria was higher (17.8%) among the school age group (6–14 years) compared to pre-school age (11.4%). Stool parasites detected included: *Entamoeba histolytica* (24/291, 8.2%), *Giardia lamblia* (13/291, 4.5%), *Hymenolepsis nana* (4/291, 1.4%), Schistosomes (3/291, 1.0%) and hookworm (1/291, 0.3%). The prevalence of malaria, HIV, helminthes and intestinal protozoa are summarised in Table 2.

**Table 2:** Prevalence of malaria, HIV, helminthes and intestinal protozoa

Variable	Preschool (1–5 years)	School (6–14 years)	Overall
Malaria^a^			
Positive	21 (11.4)	26 (17.8)	47 (14.2)
Negative	164 (88.6)	120 (82.2)	284 (85.8)
HIV^b^			
Positive	0 (0)	1 (0.7)	1 (0.3)
Negative	186(100)	145 (99.3))	331 (99.7)
Parasites^c^			
Helminthes			
Hookworm	0 (0)	1 (0.7)	1 (0.3)
*H. nana*	1 (0.7)	3 (2.1)	4 (1.4)
Schistosome	1 (0.7)	2 (1.4)	3 (1.0)
Protozoa			
*Giardia lamblia*	9 (6.0)	4 (2.8)	13 (4.5)
*Entamoeba histolytica*	13 (8.7)	11 (7.7)	24 (8.2)

Note: ^a^11, ^b^10, ^c^45 reported

Overall, anaemia prevalence was 34.4%. Anaemia was more prevalent in the age group 1–5 years (37.2%, Hb < 11.0 g/dl) compared to age group 6–11 years (33.3%, Hb < 11.5 g/dl). The lowest prevalence was found in the age group 12–14 years (11.8%, Hb < 12.0 g/dl). Some of the children, 65/329 (19.8%) had mild anaemia (Hb 10.0–10.9 g/dl) compared to 48/329 (14.6%) with moderate anaemia (Hb 8.0–9.9 g/dl). There were no cases of severe anaemia (Hb < 7.0 g/dL) in the asymptomatic children of the study population. All the nutritional deficiencies observed in this study were more common among males. This result is summarised in Table 3.

**Table 3:** Prevalence of under - nutrition

	Underweight	Stunting	Wasting
Age			
Pre-school: 1–5	21 (10.7)	32 (16.3)	10 (5.1)
School: 6–14	9 (6.2)	18 (12.3)	3 (2.6)
Sex			
Male	20 (10.9)	29 (15.9)	10 (5.8)
Female	10 (6.3)	21 (13.2)	3 (2.2)

Children who were stunted had 2.5 times risk of anaemia than non-stunted children (95% CI = 1.3, 4.7). The risk of anaemia was 2.6 times among underweight children (95% CI = 1.2, 5.6). Anaemia was also observed to decrease with increasing maternal age. Children whose mothers were between 30 and 33 years had the least risk of anaemia (OR = 0.4, 95% CI = 0.2, 0.9). Maternal parity ≥5 was negatively associated with anaemia in the offspring (OR = 0.5, 95% CI = 0.3, 0.8). Though not of statistical significance (*P = .16)*, the risk of anaemia was observed to decrease with increasing age and the least risk of anaemia was among children aged 12–14 years (OR =0.3, 95% CI = 0.1, 1.1). Boys were 1.3 times more likely to be anaemic than girls (95% CI =0.8, 2.1). Children with only one parent surviving were 1.5 times more likely to be anaemic (95% CI = 0.6, 3.9) compared to those with both parents. The risk of anaemia was 1.5 times (95% CI = 0.8, 2.9) higher among malaria positive children compared to malaria negative. Distance of the child’s home from a perennial water source (*X* ^*2*^ *= 1.3900, P = .24*), access to safe water (Fisher’s exact = .55), presence of toilet/latrine for family use and helminthes infection were not associated with anaemia in the bivariate analysis. The risk of anaemia decreased with increase in household income, and household size. There was a positive correlation between household size and income (*Pearson X* ^*2*^ *= 22.96; P = .001)*, implying that large families were of higher socioeconomic status. It is important to note that this community has a reasonable percentage of Muslims – with small businesses but also characterised by large families. Though, not of statistical significance *(P = .61)*, the risk of anaemia was found to be low among children who lived in households that reported bed net use (OR = 0.8, 95% CI = 0.3, 2.0). Anaemia was prevalent in households that consumed fewer meals per day and, without meat and fish. These results are shown in the Table 4.

**Table 4:** Bivariate Analysis of factors associated with anaemia

Variables	Have anaemia		Odds ratio (95% CI)	*P*-value
	Yes	No		
Sex				
Female^a^	48 (31.0)	107 (69.0)	1	0.224
Male	65 (37.4)	109 (62.6)	1.3 (0.8,2.1)	
Age group				
1–5^a^	68 (37.2)	128 (62.8)	1	0.156
6–11	43 (33.3)	86 (66.7)	0.9 (0.6,1.5)	
12–14	2 (11.8)	15 (88.2)	0.3 (0.1,1,1)	
Malaria				
Negative^a^	93 (32.8)	191 (67.2)	1	0.190
Positive	20 (42.6)	27 (57.4)	1.5 (0.8,2.9)	
Bed net				
No^a^	8 (38.1)	13 (61.9)	1	0.612
Yes	105 (32.7)	216 (67.3)	0.8 (0.3,2.0)	
Meat				
Never^a^	28 (34.6)	53 (65.4)	1	0.943
Once/week	44 (32.3)	92 (67.7)	0.9 (0.5,1.6)	
> 1 per week	41 (32.8)	84 (67.2)	0.9 (0.5,1.7)	
Fish				
Never^a^	18 (41.9)	25 (58.1)	1	0.336
Once/week	43 (29.9)	101 (70.1)	0.6 (0.3,1.2)	
> 1 per week	52 (33.6)	103 (66.4)	0.7 (0.3,1.4)	
Meals				
once/twice^a^	15 (36.6)	26 (63.4)	1	0.608
Thrice	98 (32.6)	203 (67.4)	0.8 (0.4,1.7)	
Survival				
Both alive^a^	105 (32.5)	218 (67.5)	1	0.388
One parent	8 (42.1)	11 (57.9)	1.5 (0.6,3.9)	
HH size (members)				
1–3^a^	19 (55.9)	15 (44.1)	1	0.021
4–6	44 (31.9)	94 (68.1)	0.4 (0.2,0.8)	
6 or more	50 (31.9)	107 (68.1)	0.4 (0.2,0.8)	
Income				
10 k–149 k^a^	31 (44.3)	39 (55.7)	1	0.136
150 k–299 k	35 (31.0)	78 (69.0)	0.6 (0.3,1.1)	
300 k–599 k	40 (30.8)	90 (69.2)	0.6 (0.3,1.0)	
600 k +	7 (24.1)	22 (75.9)	0.4 (0.1,1.1)	
Maternal parity				
1–4^a^	89 (37.9)	146 (62.1)	1	0.005
5 +	24 (22.4)	83 (77.6)	0.5 (0.3,0.8)	
Maternal age				
16–23^a^	34 (42.0)	47 (58.0)	1	0.040
24–29	39 (37.9)	64 (62.1)	0.8 (0.5,1.5)	
30–33	17 (24.3)	53 (75.7)	0.4 (0.2,0.9)	
34–58	23 (26.1)	65 (73.9)	0.5 (0.3,0.9)	
Underweight (Weight for age Z scores)				
No^a^	97 (32.2)	203 (67.7)	1	0.014
Yes	16 (55.2)	13 (44.8)	2.6 (1.2,5.6)	
Stunting (Height for age Z scores)				
No^a^	87 (31.1)	193 (68.9)	1	0.003
Yes	26 (53.1)	23 (46.9)	2.5 (1.3,4.7)	
Wasting (Weight for Height Z scores)				
No^a^	105 (36.6)	182 (63.4)	1	0.670
Yes	4 (30.8)	9 (69.2)	0.8 (0.2,2.6)	
Mother’s Highest Level of education				
No formal education^a^	12 (32.4)	25 (67.6)	1	0.576
Primary	70 (36.7)	121 (63.4)	1.2 (0.6,2.6)	
Secondary and above	31 (30.7)	70 (69.3)	0.9 (0.4,2.1)	
Father’s Highest Level of education				
No formal education^a^	4 (33.3)	8 (66.7)	1	0.926
Primary	39 (33.0)	79 (67.0)	1.0 (0.3,3.5)	
Secondary and above	70 (35.2)	129 (64.8)	1.1 (0.3,3.7)	
Presence of worms				
Yes^a^	1 (12.5)	7 (87.5)	1	0.193
No	179 (65.3)	95 (34.7)	0.27 (0.03,2.24)	

Note:^a^ (Reference group)

Stunting [(OR = 2.5, 95% CI = 1.3, 4.7), *P = .004*], household size [(OR = 0.4, 95% CI = 0.2, 0.8), *P = .021*] and maternal parity [(OR = 0.4, 95% CI = 0.3, 0.7), *P = .002]* were significantly associated with risk of anaemia. Children who were stunted were 2.5 times likely to have anaemia compared to normal children. Households with family members exceeding three had less risk of anaemia. Similarly, maternal parity ≥5 was associated with a lower risk of anaemia in the offspring. Table 5 shows the multivariate analysis of the factors associated with anaemia.

**Table 5:** Multivariate analysis of factors associated with anaemia

Variables	Have anaemia		Unadjusted Odd ratio (95% CI)	*P*-value	Adjusted Odd ratio (95% CI)	*P*-value
	Yes	No				
Sex						
Female^a^	48 (31.0)	107 (69.0)	1	0.224		
Male	65 (37.4)	109 (62.6)	1.3 (0.8,2.1)			
Age group						
1–5^a^	68 (37.2)	128 (62.8)	1	0.156		
6–11	43 (33.3)	86 (66.7)	0.9 (0.6,1.5)			
12–14	2 (11.8)	15 (88.2)	0.3 (0.1,1,1)			
Malaria						
Negative^a^	93 (32.8)	191 (67.2)	1	0.190		
Positive	20 (42.6)	27 (57.4)	1.5 (0.8,2.9)			
HH size (members)						
1–3^a^	19 (55.9)	15 (44.1)	1	0.021		
4–6	44 (31.9)	94 (68.1)	0.4 (0.2,0.8)			
6 or more	50 (31.9)	107 (68.1)	0.4 (0.2,0.8)			
Income						
10 k–149 k^a^	31 (44.3)	39 (55.7)	1	0.136		
150 k–299 k	35 (31.0)	78 (69.0)	0.6 (0.3,1.1)			
300 k–599 k	40 (30.8)	90 (69.2)	0.6 (0.3,1.0)			
600 k +	7 (24.1)	22 (75.9)	0.4 (0.1,1.1)			
Maternal parity						
1–4^a^	89 (37.9)	146 (62.1)	1	0.005	1	0.002
5 +	24 (22.4)	83 (77.6)	0.5 (0.3,0.8)		0.4 (0.3,0.7)	
Maternal age						
16–23^a^	34 (42.0)	47 (58.0)	1	0.040	1	0.321
24–29	39 (37.9)	64 (62.1)	0.8 (0.5,1.5)		1.0 (0.9,1.0)	
30–33	17 (24.3)	53 (75.7)	0.4 (0.2,0.9)			
34–58	23 (26.1)	65 (73.9)	0.5 (0.3,0.9)			
Underweight (Weight for age Z scores)						
No^a^	97 (32.2)	203 (67.7)	1	0.014	1	0.352
Yes	16 (55.2)	13 (44.8)	2.6 (1.2,5.6)		1.6 (0.6,4.2)	
Stunting (Height for age Z scores)						
No^a^	87 (31.1)	193 (68.9)	1	0.003	1	0.004
Yes	26 (53.1)	23 (46.9)	2.5 (1.3,4.7)		2.5 (1.3,4.7)	
Presence of worms						
No^a^	179 (65.3)	95 (34.7)	1	0.193	1	0.167
Yes	1 (12.5)	7 (87.5)	0.27(0.03,2.24)		0.22 (0.26,1.88)	

Note:^a^ (Reference group)

## Discussion

Our study to the best of our knowledge was the first to describe prevalence of childhood anaemia and it is risk factors in an urban setting in the North-western part of Uganda. In these settings, the overall prevalence of anaemia was 34.4%, which was higher than previously reported in similar studies conducted in East Africa [13, 17]. The prevalence of anaemia was 37.2 and 30.8% among children 1–5 and 6–14 years respectively, suggesting unmet nutritional supplements among males and young children with increased activity and growth demands, respectively. Most of the children had mild anaemia 65 (19.8%), while 48 (14.6%) had moderate anaemia. There was no case of severe anaemia in this study, which was expected especially that this was a community and not hospital based study. The 37.2% anaemia prevalence among children 1–5 years is lower than previous findings elsewhere in Uganda [16], possibly because of differences in the settings and malaria seasonality. We found lower prevalence of malaria (14.2%), compared to 60.6 and 47.5% reported by Green et al. [16] among children living along shores of Lake Albert and in the Islands of Lake Victoria where prevalence of anaemia was 68.9 and 27.3% respectively. In the same report, the prevalence of Schistosomiasis was 45.9 and 40.7% respectively [16], confirming that malaria, helminthiasis and the co-infections thereof, are key risk factors for anaemia, possibly by virtue of haemolysis, nutrient depletion, bone marrow suppression or chronic disease, or various combinations of underlying mechanisms. Nevertheless, a higher prevalence of anaemia than in our study was previously reported among school children (age 6–14 years) in two Ugandan studies [3, 4]. The discrepancy may be attributed to the difference in the study settings where by the latter were carried out in rural areas and the timing of these studies in which ours was conducted at the time when there was increased use of insecticide-treated bed nets and regular use of antihelminthes.

Malaria has been shown to cause anaemia in several studies [17, 21–24]. In our study, the prevalence of malaria is 14.2% lower than reported by Marcelline et al.*,* [17] among Rwandan children aged 1–15 years. Our study also revealed that children who had malaria were 1.5 times more likely to have anaemia compared to those who tested negative (OR = 1.5, 95% CI = 0.8, 2.9). However, this association was not statistically significant (*P = .19)* in the multivariate analysis possibly due to the low prevalence of malaria.

Helminthiasis has been shown to significantly contribute to the problem of anaemia [17, 25–27]. In our study, we did not find statistical relationship between helminthes infections and anaemia (*P = .17)* possibly due to the extremely low prevalence. Generally, Uganda has recorded tremendous progress in eliminating neglected tropical diseases over the past decade. The prevalence of Schistosomiasis declined from 42.4 to 17.9% in 2005, while hookworm prevalence reduced from 50.9 to 10.7% during the same period [28]. Other helminthes infections that showed decline were Ascariasis and Trichuriasis from 2.8 and 2.2% in 2003, to very undetectable levels in 2005 [28]. Therefore, the very low prevalence of helminthiasis in our study is not surprising.

Similarly, HIV infection in our study was very low (0.3%) and insignificant to assess the relationship with anaemia. Elsewhere, HIV infection has been associated with risk of anaemia. In two studies conducted in Uganda, the prevalence of anaemia among HIV infected children ranged from 85 to 91.7% and HIV was independently associated with anaemia [29, 30]. However, another study in Uganda found no significant difference in the prevalence of anaemia among HIV infected and uninfected children [31]. There have been pronounced improvements in the HIV care and these have reduced new HIV infections tremendously, particularly mother-to-child transmissions. The current HIV prevalence among children aged 5 years and below is less than 1% [32] and with the current strategy of EMTCT, this is likely to shrink further in the coming years.

In our study, older children (6–11 and 12–14 years) were less likely to be anaemic than younger children (1–5 years) [(OR = 0.3, 95% CI =0.1, 1.1), *P = .16*]. This finding is consistent with the previous reports that anaemia is common among children around the time of the growth spurt [33, 34]. During this period, children’s physical development is rapid, and the blood volume is largely expanded, whereas the iron storage from the maternal source has usually been depleted; diet becomes a vital source for iron as a result [35]. In a more recent community pilot survey conducted in Arua district (same study area) hemoglobin <11.0 g/dl was observed in nearly half of the children less than 16 years enrolled (unpublished data).

Anaemia was also found to be more common among male children compared to females, though, not significant [(OR = 1.3, 95% CI = 0.8, 2.4), *P = .22*]. This result is comparable with one reported by Ngesa and Mwambi [13], where the risk of anaemia was 1.2 times higher among boys than girls. The higher prevalence of anaemia in males is related to the higher growth rate in boys, resulting in greater need for iron by the body, not supplied by the diet [36].

In our study, higher maternal parity (≥5 child births) was negatively associated with anaemia (*P = .002)* contrary to the finding by Cardoso et al.*,* [37]. Elsewhere, early motherhood has also been shown to increase the risk of anaemia in the offspring [38]. The risk of anaemia in children of teenage mothers suggests that they are less prepared to meet the nutritional needs of their children and to perform the duties of motherhood. It is interesting to report that the risk of anaemia decreased with increasing household size (*P = .02*) contrary to the previous report [39]. We therefore concluded that this finding was a factor of household income rather than actual household size. In addition to the household size, we observed that the risk of anaemia declined with increasing household income. This finding is consistent with the previous reports that high socioeconomic status is associated with better nutrition, education and life [40].

The association between mother’s education level and the care provided for children has been greatly discussed in the literature, given that education has a relationship with the capacity to grasp the knowledge needed for adequate healthcare and nutrition for children, just as it provides a chance to enter the labor market and probably better socioeconomic conditions [39, 41, 42]. The results from the current study reflect this relationship, though, not significant in the bivariate analysis (*P = .58)*. The findings of this study indicate that promoting maternal health and providing mothers with anaemia - related information may help with controlling anaemia incidence. Children with a single parent surviving were 1.5 times more likely to be anaemic compared to those with both parents though this association was not statistically significant (*P = .39)*. This result is consistent with that of a study carried out in India [43].

Stunting, a proxy for chronic under-nutrition, was significantly associated with anaemia (*P = .004)*. This finding is consistent with reports elsewhere [44, 45]. Children who were stunted had 2.5 times risk of anaemia (95% CI = 1.3, 4.7). Therefore, stunting which is a consequence of malnutrition is a significant risk factor for anaemia. In our study, prevalence of underweight, stunting and wasting among children aged 1–5 years were 10.7, 16.3 and 5.1% respectively. Among children aged 6–14 years, underweight was 6.2%, stunting 12.3% and wasting 2.6%. By WHO classification [46] of public health importance of prevalence of malnutrition, our findings can be described as acceptable.

We acknowledge that resources limited the scope of this study to one geographical region, and, thus recommend similar studies in other geographical regions to confirm our findings about the changing context of nutrition, hygiene, and maternal wealth. Limited laboratory resources precluded examination of factors like hemoglobinopathies or lead intoxication, however, these factors are likely to be more important for a hospital-based study of children with severe anemia than a community-based study.

## Conclusions

Our study demonstrates that anaemia is more prevalent in under - 5 year old children. Factors independently associated with the risk of anaemia included child stunting and low maternal parity. The lower prevalence of malaria and helminthiasis in this study population suggest increased protection from the risk of anaemia in the community. Further studies are required to investigate the association between maternal parity and anaemia found in this study.
